# LONELINESS IN LGBTQIA+ OLDER ADULTS: A SCOPING REVIEW

**DOI:** 10.1093/geroni/igac059.3035

**Published:** 2022-12-20

**Authors:** Katherine Bowers, Joan Carpenter, Marleen Thornton, Hannah Fetting, Erin Johnson

**Affiliations:** University of Maryland, Baltimore, Maryland, United States; University of Maryland School of Nursing, Baltimore, Maryland, United States; Notre Dame of Maryland University, Baltimore, Maryland, United States; Johns Hopkins Bayview, Baltimore, Maryland, United States; Johns Hopkins Hospital, Baltimore, Maryland, United States

## Abstract

Loneliness in older adulthood is responsible for poor mental and physical health, cognitive decline, and early mortality. Older adults in the LGBTQIA+ community experience additional layers of vulnerability, such as discrimination, higher rates of childlessness, health inequity, and economic disparity that place them at high risk for loneliness. The purpose of this scoping review is to explore the existing literature on loneliness in LGBTQIA+ older adults and identify knowledge gaps to inform future research recommendations. Using the Arskey & O’Malley methodological framework, our team searched PubMed, Embase, CINAHL, PsychINFO, Scopus, and Google Scholar using keywords related to older adults, sexual and gender minorities, and loneliness. We identified 61 articles from 1980 to 2022. Most of the research in this literature reflected studies using descriptive and qualitative approaches. Intervention studies were the least reported. One opinion piece is included. Initial findings highlight associations between discrimination and loneliness, poor mental health and loneliness, the challenges of HIV in older adulthood and loneliness, and loneliness experienced in caregiving and end of life in LGBTQIA+ older adults. Importantly, many studies emphasized the protective nature of strong social support and community ties against loneliness. Equally, the literature suggests that limited social support is a significant risk factor for loneliness in this population. Older gender non-binary and LGBTQIA+ people of color were underrepresented in many studies. Future research should include intervention research that targets loneliness in this population as well as studies focused on understanding loneliness more comprehensively in underrepresented LGBTQIA+ older adults.

